# Spontaneous Ventral Spinal Epidural Hematoma in an Infant: An Unusual Presentation

**Published:** 2013

**Authors:** Asad ABBAS, Kamran AFZAL, Athar Abdul MUJEEB, Tabassum SHAHAB, Mohammad KHALID

**Affiliations:** 1 Registrar, Department of Pediatrics, Jawaharlal Nehru Medical College, Aligarh Muslim University, Aligarh, UP, India; 2 Associate Professor, Department of Pediatrics, Jawaharlal Nehru Medical College, Aligarh Muslim University, Aligarh, UP, India; 3 Registrar, Department of Pediatrics, Jawaharlal Nehru Medical College, Aligarh Muslim University, Aligarh, UP, India; 4 Professor, Department of Pediatrics, Jawaharlal Nehru Medical College, Aligarh Muslim University, Aligarh, UP, India; 5 Associate Professor, Department of Radio-diagnosis, Jawaharlal Nehru Medical College, Aligarh Muslim University, Aligarh, UP, India

**Keywords:** Spinal Hematoma, Spinal Epidural Hematoma, Spontaneous Epidural Hematoma, Magnetic Resonance Imaging, Neurological Deficit

## Abstract

Spontaneous ventral spinal epidural hematomas are extremely rare in children and clinically recognized by the appearance of acute asymmetric focal motor and sensory involvement. In infants, the initial presenting symptoms are very non-specific and irritability is often the only initial manifestation. Appearance of other neurological signs may be delayed up to hours or even days later. In the absence of significant precipitating factors such as severe trauma or previously known coagulopathies, the diagnosis is usually delayed until the full picture of severe cord compression is developed. The diagnosis is finally made by performing magnetic resonance imaging. We report a 5-month-old infant with spinal epidural hematoma who presented with symmetrical upper limb weakness and diaphragmatic involvement to highlight the importance of recognizing the atypical manifestations for early diagnosis and intervention.

## Introduction

Spontaneous spinal epidural hematoma is an uncommon cause of cord compression in children. Ventral spinal hematomas are even rarer with only two cases of them being reported in the literature, to date (1,2). Although precipitating factors such as anticoagulant therapy (3), hemophilia (4), and arteriovenous malformations (5) may be present, 40-50% of the reported cases have no identifiable risk factor for the situation (3). Spontaneous cases are those in whom trivial trauma has occurred. Posttraumatic cases are relatively rare (6). The initial presentation is usually nonspecific. The most common symptom is pain manifested by irritability in the infants followed by progressive focal motor and sensory deficits due to spinal cord compression within hours or even days. We present a 5-month-old infant with ventral cervical epidural hematoma who presented with symmetrical upper limb weakness and diaphragmatic involvement. 

## Case Report

**Fig1(A, B):**
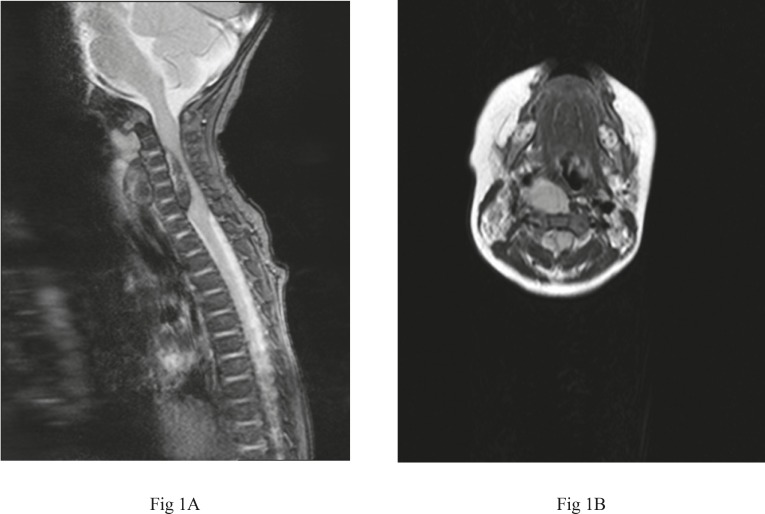
MRI of the spine showing an epidural hematoma extending from C4 to C7 leading to displacement and compression of the spinal cord with expansion of segments proximal and distal to the site as well as a hematoma in pre and paravertebral spaces on axial view. A Sagittal T2-weighted image (TR 550 ms, TE 24 ms); B Axial T2-weighted image

A 5-month-old previously healthy boy presented with the history of not moving his upper limbs, crying on handling, and weak cry. Twenty-four hours prior to the onset of these symptoms the patient had fallen from a low bed (40 cm) to a carpeted floor during his night sleep. The parents denied head trauma or neck twist. He was well and active in the intervening periods with no feeding problems. There was no personal or family history of a bleeding diathesis or anticoagulation treatment. The infant had never undergone any invasive spinal procedure including spinal tap and no history of prolonged bleeding following injuries including circumcision was present, either. On admission, the patient was afebrile, awake, and alert. However, he was irritable and cried while being handled. Neck movements seemed painful but without meningismus. The cervical spine was therefore immobilized. No abnormality was found on local examination of the neck. Neurological examination revealed symmetrical flaccid weakness in both upper limbs at shoulder, elbow, and wrists with maximum power of 1/5. Pain sensation appeared to be lost in both upper limbs and deep tendon reflexes were not elicitable. In the lower extremities, there was no motor weakness although the reflexes were brisk with an extensor plantar response. There was no involvement of bladder and bowel functions and anal and cremasteric reflexes were preserved. The respiration appeared to be jerky and labored because of diaphragmatic involvement but oxygen saturation was normal. The rest of the examination was normal. His hemogram was normal (total leukocyte count was 8700 with 65% neutrophils and 37% lymphocytes), serum sodium was 139 mEq/L, and serum potassium was 4.2 mEq/L. Coagulation studies showed a prothrombin time of 22 seconds (normal range; 10-13 seconds) with an INR of 2.1 that normalized after one dose of vitamin K. The activated partial thromboplastin time was normal 25 seconds (normal range; 25-40 seconds), platelet count was 185,000/μL and bleeding time was1 minute and 20 seconds. Plain radiographs of the spine and chest were normal and demonstrated no spinal fracture or elevation of diaphragm. Nerve conduction velocities were also normal in all limbs. MRI of the spine was performed on day 2 of admission which demonstrated a ventral epidural hematoma extending from C4 to C7 vertebral levels leading to severe compression and displacement of the cervical cord towards left side with mild expansion of the cord proximal and distal to the compressed segment suggestive of edema or infarction. Another hematoma, a possible extension from epidural space, was present in the pre and paravertebral spaces with extension along posterior cervical and parapharyngeal spaces of the neck (Fig.1). The patient was referred to a neurosurgery referral hospital for urgent surgical decompression. 

## Discussion

Spontaneous spinal epidural hematoma (SSEH) is an idiopathic accumulation of blood in the vertebral epidural space. Spinal epidural hematomas are extremely rare in children. The annual incidence of SSEH is almost 0.1 per 100,000 patients in the general population. This is while this incidence is significantly lower in pediatric population (7). It is usually confined to the dorsal epidural space. Ventral SSEH, as our case, is even rarer with only two previous reports of it being available in the literature (1,2). The dural sac is attached to the spinal bony canal by connective tissue strands on the ventral surface while the space is filled by fatty tissue on the dorsal surface. No sex preponderance has been defined in the situation and the presenting age ranges from in utero to 14 years (8,9).

The most common site in children, as recently documented in 27 cases, is C-5 to T-1 (2). The site of involvement in our case was slightly higher involving C-4, as well, leading to involvement of diaphragm to a greater extent than what has been reported so far. 

The clinical presentation of spinal epidural hematomas in pediatric patients varies significantly. Pain is usually the first symptom which can be localized to the level of hematoma or present as a radicular pain. It manifests with abnormal crying in infants making the appropriate clinical localization of the lesion even more challenging. The progression of the symptomatology and clinical signs is usually very rapid, as in our case, although slower progression over a few days has also been reported (10). Clinical presentation was different in our patient. Neurological deficit in upper limbs was bilateral and strikingly symmetrical with flaccidity and apparent loss of sensations led us to an initial suspicion of a demyelinating polyneuropathy like Guillian Barrĕ syndrome. 

In only 50-60% of cases a cause for hematoma can be elucidated, the most common of which, are use of anticoagulants (3), coagulopathies (either congenital such as hemophilia (4) or acquired such as leukemia), and procedures such as spinal tap or epidural anesthesia (11). Trauma is surprisingly rare in children with few cases of it being described (6). Cases without known predisposing factors are known to be spontaneous. Cases with minor traum are also described as spontaneous (12,13). Since our patient’s symptoms developed after he had fallen from a low bed onto a carpeted floor and because falls of this kind are very common in the pediatric population and rarely associated with clinical symptoms, we suggested a diagnosis of SSEH. 

The pathogenesis of SSEH is unclear; however, bleeding is believed to be venous in origin. Lack of valves in the epidural venous plexus makes it especially vulnerable to any intrinsic change in pressure. Whooping cough (12) and activities such as voiding (13), which can cause prolonged valsalva and sudden fluctuation in intra-abdominal and intrathoracic pressure, have been described preceding the neurological symptoms. Other theories, such as the idea that epidural arterial hemorrhage causes mechanical disruption and traction on nerve roots or spontaneous rupture of occult arteriovenous malformation (5), have also been suggested. No such malformations were detected on our patient’s MRI. 

Urgent surgical decompression and evacuation of the epidural hematoma is the treatment of choice. Complete and partial neurological recovery has been described in approximately 50% and 44% of the patients, respectively (14). Recovery was significantly better when decompression was performed within less than 36 hours of the onset of the neurological deficit. Children may have a better potential for recovery compared to the adults. A high index of suspicion can lead to an early diagnosis and intervention.
